# From Dry Box to Dry Cup: Portable Laparoscopic Training Through Task Decomposition

**DOI:** 10.7759/cureus.107119

**Published:** 2026-04-15

**Authors:** Takuma Iwai, Hiroshi Makino, Hiroshi Yoshida

**Affiliations:** 1 Gastroenterological Surgery, Nippon Medical School, Tokyo, JPN

**Keywords:** education and training, laparoscopic box training, laparoscopic surgeries, laparoscopic training, skill training

## Abstract

Laparoscopic training is prolonged because it requires adaptation to indirect two-dimensional vision and hand-eye dissociation. Yet, access to effective off-the-job training remains uneven because of limited time, space, mentorship, and simulation resources. We propose the “Dry Cup” as a portable, decomposition-first approach to laparoscopic training rather than a standardized device. Using readily available materials, short portable instruments, a webcam, and consumer-grade display devices, the Dry Cup creates a portable two-dimensional training environment outside dedicated simulation spaces. Complex laparoscopic skills are decomposed hierarchically into trainable work units, allowing learners to localize the true bottleneck of performance and practice specific components in short, repeated sessions. In this framework, portability applies not only to the physical setup but also to the visual environment. The Dry Cup is a portable training philosophy that enables task-specific practice anytime and anywhere. By linking portability with hierarchical task decomposition, this approach may help integrate deliberate off-the-job training into daily clinical life and reduce disparities in laparoscopic education, particularly in resource-limited settings.

## Introduction

Laparoscopic surgery has become a standard approach in gastrointestinal surgery owing to its minimally invasive nature, magnified operative view, and ease of sharing surgical videos. Despite these advantages, laparoscopic procedures impose unique cognitive and motor demands, including adaptation to two-dimensional vision and coordination between indirect visual input and hand movements. Consequently, skill acquisition often requires substantially longer training periods than open surgery.

In Japan, the Endoscopic Surgical Skill Qualification System administered by the Japan Society for Endoscopic Surgery aims to standardize laparoscopic competence. Nevertheless, certification remains demanding, and successful candidates are often concentrated in high-volume centers. Similar disparities are observed worldwide, reflecting broader limitations in access to training resources, mentorship, and time.

Concurrently, reforms aimed at reducing excessive working hours for physicians have further constrained opportunities for in-hospital training. Under these conditions, surgical education must be redesigned to achieve maximum educational effect with minimal time and resource expenditure. Educational research indicates that improvement is driven not by practice volume alone, but by deliberate practice, in which the training target is explicitly defined, and feedback is tightly linked to performance outcomes. In surgical education, deliberate practice has been shown to improve technical performance and quality compared with unstructured repetition or experience-based learning alone [1-3]. Recent systematic reviews have further highlighted the role of deliberate practice in enhancing simulation-based laparoscopic training outcomes [4]. However, existing training frameworks do not always specify how to identify the most appropriate unit of practice within a complex surgical task.

Based on this framework, we propose a portable training philosophy that moves the training environment beyond dedicated simulation spaces. Rather than increasing the volume of undifferentiated practice, this approach redefines the unit of practice through task decomposition, allowing surgeons to identify the true performance bottleneck within complex surgical tasks. Its feasibility is further supported by the widespread availability of smartphones and consumer-grade digital devices, which make portable two-dimensional visual environments readily achievable. This technical report describes a decomposition-first, portable approach to laparoscopic training designed to address these contemporary constraints.

## Technical report

Educational concept: decomposition-first training design

**Figure 1:**
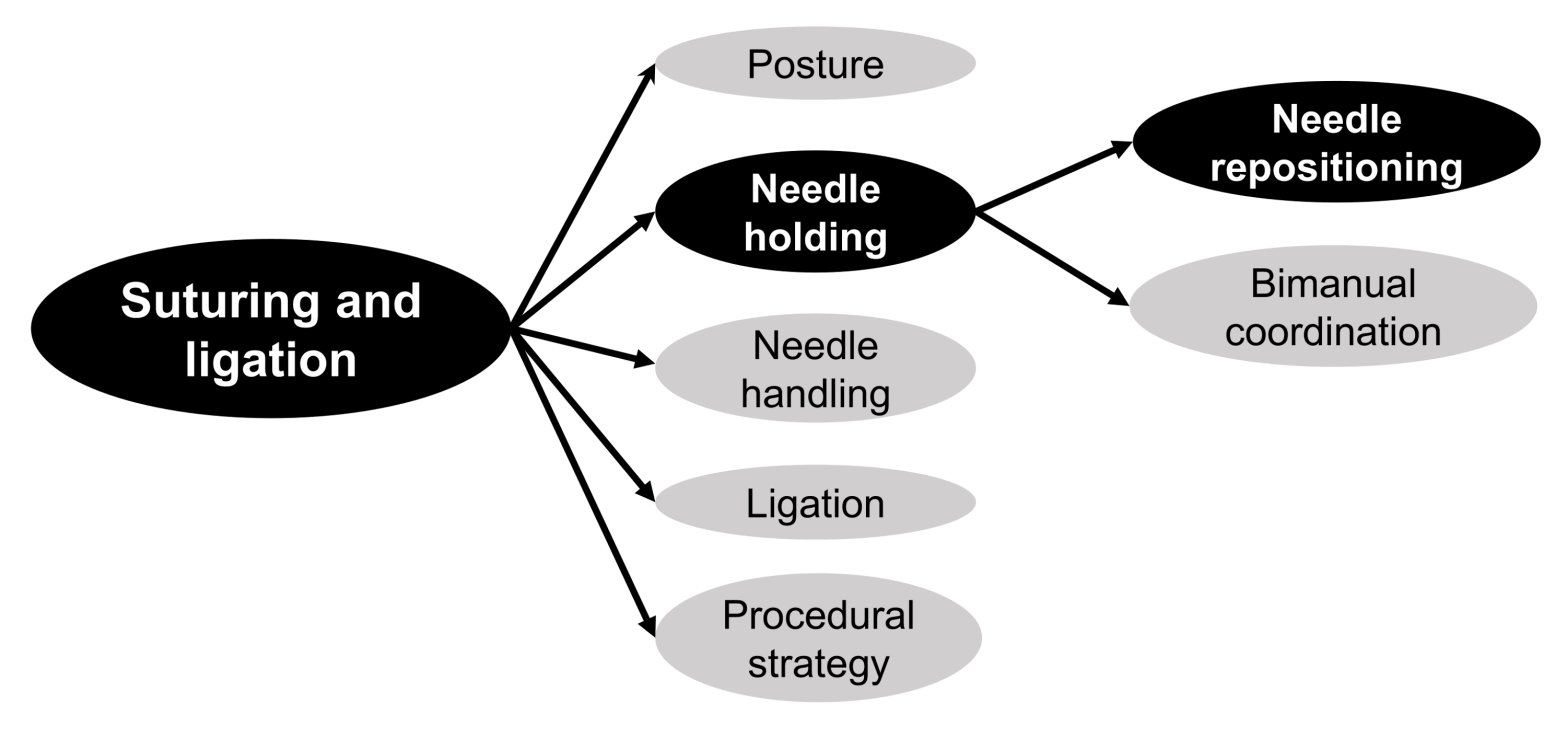
Hierarchical task decomposition of suturing and ligation. An illustrative decomposition map showing how a complex laparoscopic task can be broken down into progressively narrower work units. In this example, the learner’s main bottleneck is localized hierarchically from suturing and ligation to needle holding, and further to needle repositioning. This framework allows training targets to be identified at the level of the actual performance deficit. Created by the authors using Microsoft PowerPoint.

We define decomposition as the process of converting a complex surgical procedure into explicit, independent sub-tasks that can be trained individually. Decomposition operates along two axes: 1. Procedure-level decomposition, in which an operation is divided into reproducible steps; and 2. Action-level decomposition, in which each step is further divided into the micro-skills required from the operator and assistant. Importantly, the value of this framework is not merely descriptive; it allows the learner’s true bottleneck to be localized progressively from the overall task to a narrower work unit. In the example shown in Figure 1, the training target is narrowed from suturing and ligation to needle holding, and finally to needle repositioning.

When training is performed without distinguishing these units, learners tend to repeat the entire task indiscriminately, resulting in inertial repetition and early learning plateaus. In contrast, decomposition enables deliberate practice at the level of the actual performance deficit. This approach is consistent with prior work supporting structured simulation, proficiency-based training, and objective performance assessment in laparoscopic surgery [5-7].

The Dry Cup concept and construction

The Dry Cup was conceived not as a standardized simulator to be reproduced in a fixed manner, but as a portable training philosophy that demonstrates how a functional training environment can be constructed outside dedicated simulation spaces. In this report, portability refers not only to compact physical size, but also to the ability to externalize and carry the training environment itself into ordinary workspaces. Simulation-based training improves laparoscopic skill acquisition, but access to dedicated skills laboratories and commercial simulators remains uneven, particularly in resource-limited settings [8].

**Figure 2:**
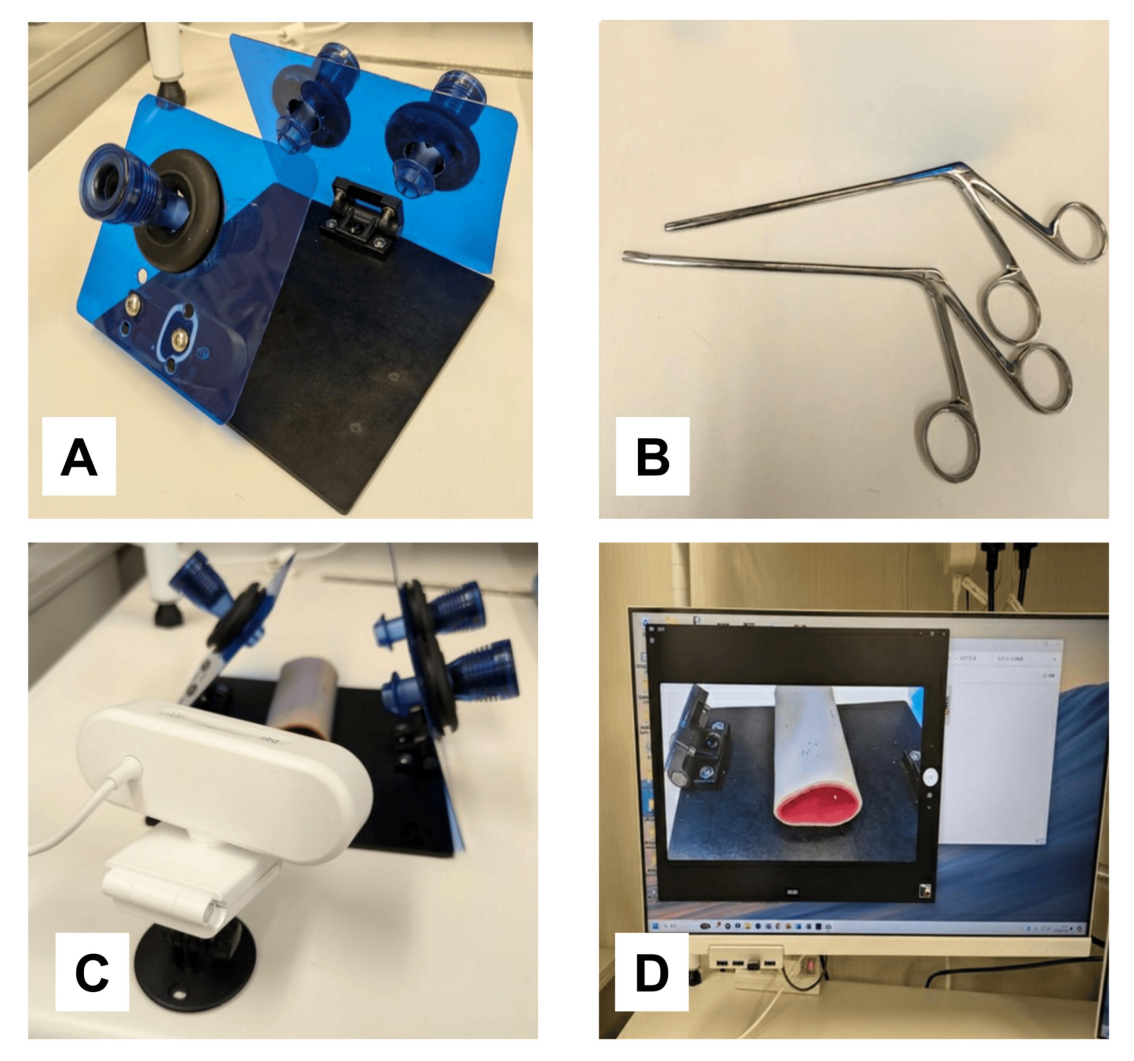
Portable physical and visual training environment. (A) Example configuration of a Dry Cup platform constructed using commonly available materials. (B) Short, angled alligator forceps used for decomposition-based practice while preserving portability. (C) A consumer-grade web camera positioned to capture the working field. (D) Real-time two-dimensional visual output displayed on a standard monitor. Together, these components illustrate one possible portable training environment for practicing decomposed laparoscopic tasks independent of specialized infrastructure.

The sample configuration shown in Figure 2 and Video 1 uses simple, readily available materials, including acrylic boards, a rubber mat, and hinges. The hinged structure provides both base stability and adjustable board angles. In the present model, acrylic boards are mounted on both sides to allow different task settings, including mirror-image practice. This configuration is presented as one possible example rather than a prescribed design; the exact setup may be modified according to the learner’s specific training needs.

**Video 1:** Demonstration of the Dry Cup portable training environment. This video demonstrates one possible configuration of the Dry Cup portable training environment using simple, readily available materials. It shows the physical setup, the creation of a portable two-dimensional visual environment using a web camera and a smartphone or computer, and an example of decomposition-based task practice. The Dry Cup is presented not as a standardized device to be replicated, but as a portable training philosophy that enables task-specific off-the-job training. The video was created by the authors.

This approach aligns with evidence indicating that low-cost and portable training systems can provide meaningful educational value when training objectives are clearly defined [9-11].

Visual portability and two-dimensional configuration

A defining feature of laparoscopic surgery is reliance on indirect, two-dimensional visual input. The Dry Cup, therefore, incorporates visual portability in addition to physical portability. Using a consumer-grade web camera and a smartphone or personal computer, the working field can be converted into a portable two-dimensional visual environment without the need for dedicated endoscopic imaging systems.

Training environments that reproduce this perceptual constraint are relevant to skill development. Prior studies have shown that simulation-based camera navigation training improves technical skills; one randomized trial improved camera navigation skills without demonstrating clinical transfer, whereas another demonstrated transfer to the operating room and greater time efficiency [12,13]. Video 1 demonstrates the setup of this visual environment and its use during task-specific practice.

Instrument selection and scope of use

Short, angled instruments such as otolaryngology alligator forceps were intentionally selected to preserve portability and accessibility. We acknowledge that these instruments do not reproduce the full mechanical characteristics of standard laparoscopic instruments, particularly the long shaft and abdominal wall fulcrum effect.

However, the aim of the Dry Cup is not to replicate laparoscopic surgery as a whole. Rather, it is designed as a decomposition-based training framework in which specific components of performance are isolated and practiced deliberately. Within this scope, the platform is intended to support elements such as grasping precision, needle holding, needle repositioning, and bimanual coordination. Portable and low-cost trainers have been shown to provide meaningful educational value for basic laparoscopic skill acquisition when training objectives are clearly defined [9-11]. The Dry Cup should therefore be understood as a complementary off-the-job training approach, not as a replacement for training with standard laparoscopic instruments or supervised operative experience.

Practical use and training modules

Training with the Dry Cup is performed in short, frequent sessions, each focused on a single decomposed unit. This structure is consistent with principles of deliberate practice, in which learning efficiency is maximized by repeated, goal-directed practice with immediate feedback [1-4].

A typical session begins by selecting one target component, reviewing the corresponding movement goal, and performing repeated trials while monitoring an observable success criterion, such as stable needle orientation, controlled repositioning without wobble, or consistent coordination between both hands. Representative modules include posture control, needle holding, needle handling, needle repositioning, ligation, and bimanual coordination. Learners progress to subsequent units only after achieving stable performance across multiple consecutive trials. In this way, the Dry Cup does not aim to replace whole-procedure simulation, but to make repeated practice of specific bottleneck actions feasible in a portable environment.

## Discussion

The principal contribution of the Dry Cup is not portability alone, but the explicit implementation of task decomposition as a training philosophy. Educational disparities in laparoscopic surgery are often addressed by expanding access to equipment or centralized training programs. However, evidence from surgical education suggests that how practice is structured is at least as important as where it takes place. In particular, simulation-based medical education with deliberate practice has been shown to outperform traditional clinical education for skill acquisition, and proficiency-based training emphasizes progression against explicit performance criteria rather than exposure alone [6,14].

The Dry Cup is not intended to replicate the full mechanical characteristics of laparoscopic instruments, but to enable deliberate practice of decomposed performance elements and should therefore be used as a complementary training approach. By redefining the unit of practice, decomposition allows training to focus on the true source of difficulty rather than on undifferentiated repetition. Portability then becomes a mechanism for continuity: small fragments of available time can be converted into deliberate, task-aware practice within ordinary workspaces. This logic is consistent with broader evidence showing that simulation-based laparoscopic training improves technical performance compared with no intervention and is moderately more effective than nonsimulation instruction [8].

The visual component of the Dry Cup is also relevant to this framework. By converting the working field into a two-dimensional image using widely available consumer devices, the platform preserves an important perceptual feature of laparoscopic surgery while remaining feasible outside specialized simulation facilities. Previous studies of camera-navigation training suggest that technical skills related to indirect vision can be improved in simulation settings, although transfer to the operating room is not uniform across studies [12,13].

Conceptually, the Dry Cup should therefore be understood as a portable mindset for surgical training rather than as a simulator. It does not replace supervised operating room experience or high-fidelity simulation; instead, it provides a practical framework for extending deliberate, decomposed practice into everyday clinical life, particularly in resource-limited settings. Portable and low-cost trainers have shown educational value for basic skills acquisition when training objectives are clearly defined [9-11].

This report has limitations. It does not include quantitative validation, comparative outcome data, or formal assessment of skill transfer. Accordingly, the present manuscript should be interpreted as a technical report describing a conceptual and practical training framework rather than an empirical effectiveness study. Future studies should evaluate learning outcomes using objective performance measures and validated assessment tools.

## Conclusions

We present the Dry Cup as a portable, decomposition-first approach to laparoscopic training in resource-limited settings. This framework enables task-specific off-the-job training using simple and widely available tools. Its educational value and broader impact on surgical training require further validation in future studies.

## References

[1] Ericsson KA (2004). Deliberate practice and the acquisition and maintenance of expert performance in medicine and related domains. Acad Med.

[2] Crochet P, Aggarwal R, Dubb SS (2011). Deliberate practice on a virtual reality laparoscopic simulator enhances the quality of surgical technical skills. Ann Surg.

[3] Hashimoto DA, Sirimanna P, Gomez ED (2015). Deliberate practice enhances quality of laparoscopic surgical performance in a randomized controlled trial: from arrested development to expert performance. Surg Endosc.

[4] Wickramasinghe D, Vincent J (2025). The use of deliberate practice in simulation-based surgical training for laparoscopic surgery - a systematic review. BMC Med Educ.

[5] Grantcharov TP, Kristiansen VB, Bendix J, Bardram L, Rosenberg J, Funch-Jensen P (2004). Randomized clinical trial of virtual reality simulation for laparoscopic skills training. Br J Surg.

[6] Gallagher AG, Ritter EM, Champion H (2005). Virtual reality simulation for the operating room: proficiency-based training as a paradigm shift in surgical skills training. Ann Surg.

[7] Aggarwal R, Grantcharov T, Moorthy K, Milland T, Darzi A (2008). Toward feasible, valid, and reliable video-based assessments of technical surgical skills in the operating room. Ann Surg.

[8] Zendejas B, Brydges R, Hamstra SJ, Cook DA (2013). State of the evidence on simulation-based training for laparoscopic surgery: a systematic review. Ann Surg.

[9] Hruby GW, Sprenkle PC, Abdelshehid C, Clayman RV, McDougall EM, Landman J (2008). The EZ Trainer: validation of a portable and inexpensive simulator for training basic laparoscopic skills. J Urol.

[10] Wong J, Bhattacharya G, Vance SJ, Bistolarides P, Merchant AM (2013). Construction and validation of a low-cost laparoscopic simulator for surgical education. J Surg Educ.

[11] Sellers T, Ghannam M, Asantey K, Klei J, Olive E, Roach VA (2021). An early introduction to surgical skills: validating a low-cost laparoscopic skill training program purpose built for undergraduate medical education. Am J Surg.

[12] Nilsson C, Sorensen JL, Konge L, Westen M, Stadeager M, Ottesen B, Bjerrum F (2017). Simulation-based camera navigation training in laparoscopy-a randomized trial. Surg Endosc.

[13] Franzeck FM, Rosenthal R, Muller MK (2012). Prospective randomized controlled trial of simulator-based versus traditional in-surgery laparoscopic camera navigation training. Surg Endosc.

[14] McGaghie WC, Issenberg SB, Cohen ER, Barsuk JH, Wayne DB (2011). Does simulation-based medical education with deliberate practice yield better results than traditional clinical education? A meta-analytic comparative review of the evidence. Acad Med.

